# A 3D epithelial–mesenchymal co-culture model of human bronchial tissue recapitulates multiple features of airway tissue remodeling by TGF-β1 treatment

**DOI:** 10.1186/s12931-017-0680-0

**Published:** 2017-11-22

**Authors:** Shinkichi Ishikawa, Kanae Ishimori, Shigeaki Ito

**Affiliations:** Scientific Product Assessment Center, R&D Group, Japan Tobacco Inc., 6-2 Umegaoka, Aoba-ku, Yokohama, Kanagawa 227-8512 Japan

**Keywords:** Bronchi, Transforming growth factor β1, Co-culture, Remodeling, Gel contraction

## Abstract

**Background:**

The collagen gel contraction assay measures gel size to assess the contraction of cells embedded in collagen gel matrices. Using the assay with lung fibroblasts is useful in studying the lung tissue remodeling process in wound healing and disease development. However, the involvement of bronchial epithelial cells in this process should also be investigated.

**Methods:**

We applied a layer of mucociliary differentiated bronchial epithelial cells onto collagen gel matrices with lung fibroblasts. This co-culture model enables direct contact between epithelial and mesenchymal cells. We stimulated the culture with transforming growth factor (TGF) β1 as an inducer of tissue remodeling for 21 days, and measured gel size, histological changes, and expression of factors related to extracellular matrix homeostasis.

**Results:**

TGF-β1 exerted a concentration-dependent effect on collagen gel contraction and on contractile myofibroblasts in the mesenchymal collagen layer. TGF-β1 also induced expression of the mesenchymal marker vimentin in the basal layer of the epithelium, suggesting the induction of epithelial-mesenchymal transition. In addition, the expression of various genes encoding extracellular matrix proteins was upregulated. Fibrotic tenascin-C accumulated in the sub-epithelial region of the co-culture model.

**Conclusion:**

Our findings indicate that TGF-β1 can affect both epithelial and mesenchymal cells, and induce gel contraction and structural changes. Our novel in vitro co-culture model will be a useful tool for investigating the roles of epithelial cells, fibroblasts, and their interactions in the airway remodeling process.

**Electronic supplementary material:**

The online version of this article (10.1186/s12931-017-0680-0) contains supplementary material, which is available to authorized users.

## Background

Airway remodeling is an important aspect in the pathogenesis of various lung diseases [1], and involves persistent changes in the normal architecture of airway walls. These changes include mesenchymal alterations such as increased numbers of smooth-muscle cells, myofibroblast accumulation, and increased matrix protein deposition [2, 3]. Fibroblasts embedded in 3D collagen matrices are a well-known in vitro mesenchymal model. Various studies employing this model have shown that transforming growth factor beta (TGF-β) enhances contraction of collagen gels and is involved in the remodeling process [4–6]. These findings suggest the usefulness of the 3D collagen-matrices model with lung fibroblasts in investigating the airway remodeling process in connective tissue. However, this model lacks the bronchial epithelial layer over the mesenchymal layer of airway walls.

Bronchial epithelium plays an important role in the defense against inhaled allergens, viral infections, and airborne pollutants [7]. Exposure to these materials induces injury and repair or inflammatory responses in bronchial epithelium, and constant stimulation may result in imbalances in these responses [8, 9]. The involvement of several mediators, including TGF-β, has been reported in these abnormal responses [10]. Thus both mesenchymal cells and bronchial epithelial cells participate in the airway remodeling process. Several studies have indicated the importance of the epithelial-mesenchymal trophic unit (EMTU) in the tissue remodeling process [11, 12]. EMTU consists of epithelial cells, mesenchymal cells, and their extracellular matrix (ECM), and local exchange of information between EMTU components is important in the response to various stimuli.

In this study, we investigated the effect of TGF-β1 on human bronchial tissue remodeling using our original 3D co-culture model. We applied human bronchial epithelial cells (HBECs) onto the 3D collagen matrices, and cultured the cells in an air-liquid interface (ALI), which enables differentiation of pseudostratified bronchial epithelium with goblet and ciliated cells [13]. Our previous report shows that this model has a mucociliary differentiated bronchial epithelial layer on a fibroblast-embedded mesenchymal collagen layer [14]. Various in vitro studies have reported interactions between epithelial cells and fibroblasts. However, these in vitro studies were performed with conditioned medium or indirect co-culture (e.g. floating co-culture, transmembrane co-culture) [15–17]. Different from these methods, our in vitro EMTU model reproduces direct interactions between epithelial cells, fibroblasts, and their ECM.

We analyzed the effects of TGF-β1 on collagen gel contraction and epithelial and mesenchymal cell layers in the 3D co-culture model of human bronchial tissue. Other endpoints related to ECM homeostasis were investigated, including expression of matrix metalloproteinases (MMPs), tissue inhibitor of metalloproteinase (TIMP), and ECM proteins.

## Methods

### Cell culture

Human fetal lung fibroblasts (IMR-90) were obtained from the American Type Culture Collection (Manassas, VA, USA) and grown in minimum essential medium (MEM) (Life Technologies, Carlsbad, CA, USA) with 10% fetal bovine serum (FBS; MP Biomedicals, Santa Ana, CA, USA). Normal HBECs (Lonza, Basel, Switzerland) were grown in Airway Epithelial Cell Growth Medium with SupplementPack (PromoCell, Heidelberg, Germany).

### 3D culture

The methodological details for the 3D co-culture of IMR-90 cells and HBECs were described in our previous paper [14]. Cellmatrix type I-A (Nitta Gelatin, Osaka, Japan), 10 × MEM and reconstitution buffer (Nitta Gelatin) were mixed with 8:1:1 by volume ratios and applied to cell culture insert (10.5 mm diameter, 1.0-μm pore size, BD Biosciences, Franklin Lakes, NJ, USA) in 100 μL aliquots to prepare base layer. The base layer was gelled by placing in an incubator at 37 °C with a 5% CO_2_ atmosphere for more than an hour. IMR-90 cells (approximately 2.5 × 10^6^ cells/mL in FBS), Cellmatrix type I-A, Cellmatrix type I-P (Nitta Gelatin), 10 × MEM, and reconstitution buffer were mixed with 1:4:4:1:1 by volume ratios and poured onto the base layer in 250 μL aliquots to prepare the collagen-embedded fibroblast layer. The fibroblast layer was gelled by placing in an incubator at 37 °C with a 5% CO_2_ atmosphere for more than an hour. After 2 days of cultivation with MEM containing 10% FBS, HBECs suspended in Airway Epithelial Cell Growth Medium (approximately 3.0 × 10^5^ cells/mL) were seeded onto the collagen layer to prepare the co-culture model, and cultured under submerged conditions until reaching a semi-confluent state. The fibroblast mono-culture model was prepared without seeding of HBECs. The ALI culture was then initiated to induce mucociliary differentiation. The mono-culture model was also cultivated under ALI conditions. PneumaCult-ALI medium (Stemcell Technologies, Vancouver, BC, Canada) was supplemented with heparin (Stemcell Technologies) and hydrocortisone (Stemcell Technologies) according to the manufacturer’s instructions. GM6001 (30 nM, Sigma-Aldrich, St. Louis, MO, USA) and 1% FBS were also added to prepare the ALI culture medium. The apical and basolateral media were removed, and 600 μL of ALI culture medium were added to the bottom well. Stimulation of TGF-β signaling with TGF-β1 (R&D Systems, Minneapolis, MN, USA) and inhibition of TGF-β signaling with a TGF-β receptor type I blocker (SB525334; Wako Pure Chemical Industries, Osaka, Japan) commenced on the first day of ALI culturing. The ALI culture was maintained for 21 days. Images of each collagen gel were obtained, and gel contraction was analyzed with ImageJ software (National Institutes of Health, Bethesda, MD, USA). Data are expressed as the percentage to the initial gel area.

### Histological analysis

After fixation in 4% paraformaldehyde at 4 °C on ALI culture day 21, bronchial tissue samples were embedded in paraffin and 5-μm sections were prepared using a microtome. Sections were deparaffinized and subjected to hematoxylin and eosin staining or immunostaining. Immunostaining was performed with Polink-2 Plus (GBI Labs, Bothell, WA, USA) using the following antibodies: Anti-vimentin antibody (1:250; ab92547; Abcam, Cambridge, UK), anti-alpha-smooth muscle actin (α-SMA) antibody (1:1000; ab5694; Abcam), anti-E-cadherin antibody (1:250; ab40772; Abcam), anti-acetylated α-tubulin antibody (1:250; ab24610, Abcam), anti-MUC5AC antibody (1:250; ab3649; Abcam), anti-cytokeratin (CK) 5 antibody (1:100; ab52635; Abcam), anti-fibronectin antibody (1:250; ab2413; Abcam), and anti-tenascin-C antibody (1:250; ab108930; Abcam). Sections were subjected to heat-induced antigen retrieval in a 10-mM sodium citrate buffer (pH 6.0) for α-SMA, E-cadherin, acetylated α-tubulin, MUC5AC, CK5 and fibronectin staining at approximately 95 °C for 30 min. EDTA (1 mM, pH 8.0) was used for vimentin and tenascin-C staining. Image analysis of immunostained sections was conducted with ImageJ software. Three sections were prepared in each experimental condition, and the area of the epithelial layer and mesenchymal collagen layer was measured. Colour Deconvolution plugin [18] was used for diaminobenzidine (DAB) and hematoxylin stain separation, and the DAB-positive areas in the epithelial and mesenchymal layers were measured. The results are expressed as the percentage of DAB-positive area in each layer.

### Gelatin zymography

Culture medium collected on ALI culture day 21 was subjected to sodium dodecyl sulfate-polyacrylamide gel electrophoresis in 7.5% acrylamide gels containing 0.9 mg/mL gelatin. After electrophoresis, gels were washed twice (30 min and 45 min) in wash buffer (0.5% Triton X-100, 2.5 mM Tris-HCl, 150 mM NaCl) and incubated for 19 h in incubation buffer (2.5 mM Tris-HCl, 20 mM NaCl, 10 mM CaCl_2_), then stained with 0.1% Coomassie blue. Images were obtained with ImageQuant LAS 4000 (GE Healthcare, Little Chalfont, UK), and signal densities were quantified using ImageQuant TL (GE Healthcare). Data are expressed as the fold change to the band density in the control.

### Measurement of TIMP by multi-plex assay

The concentrations of TIMPs secreted into the culture medium were analyzed with a Bio-Plex Pro Human TIMP Panel (Bio-Rad, Hercules, CA, USA) using the Bio-Plex system (Bio-Rad) according to the manufacturer’s instructions. Culture medium collected on ALI culture day 21 was diluted at 1:10,000 for TIMP-1 analysis or 1:100 for TIMP-2 analysis. The concentrations of TIMP-3 and TIMP-4 were lower than detection limits, although the culture medium was analyzed without dilution.

### PCR array

Total RNA was isolated from tissues using RNeasy (Qiagen, Hilden, Germany), and RNA quality was analyzed with an Agilent 2100 Bioanalyzer (Agilent Technologies, Santa Clara, CA, USA). The RNA integrity number of the samples was ≥7.6. cDNA was synthesized with a High-Capacity cDNA Reverse Transcription Kit (Applied Biosystems, Waltham, MA, USA). The gene expression profile was analyzed with the Human Extracellular Matrix and Adhesion Molecules RT 2 Profiler PCR Array (PAHS-013, SABiosciences, Frederick, MD, USA) on an ABI 7900 PCR system (Applied Biosystems).

### Statistical analysis

With the exception of the PCR array results, data are presented as the means and standard deviations of triplicate inserts. Multiple-comparisons tests were used in the analysis of the results obtained with co-culture model. Bartlett’s test was used to confirm homogeneity of variances from multiple groups. A parametric one-way analysis of variance followed by Dunnett’s test was performed to detect statistically significant differences. Results were considered significant at *p* < 0.05. For the analysis of the fibroblast mono-culture model, student’s *t*-test was used and the results were considered to be significant at *p* < 0.05. All statistical analyses except for those of PCR array data were performed using Ekuseru-Toukei (SSRI Co., Ltd., Tokyo, Japan). The PCR array data were analyzed using RT 2 Profiler PCR Array Data Analysis version 3.5, provided by SABiosciences. The results are presented as the means and 95% confidence intervals of the triplicate inserts. Student’s *t*-test was used; the results were considered significant at *p* < 0.05.

## Results

### Collagen gel contraction following TGF-β1 stimulation

**Fig. 1:**
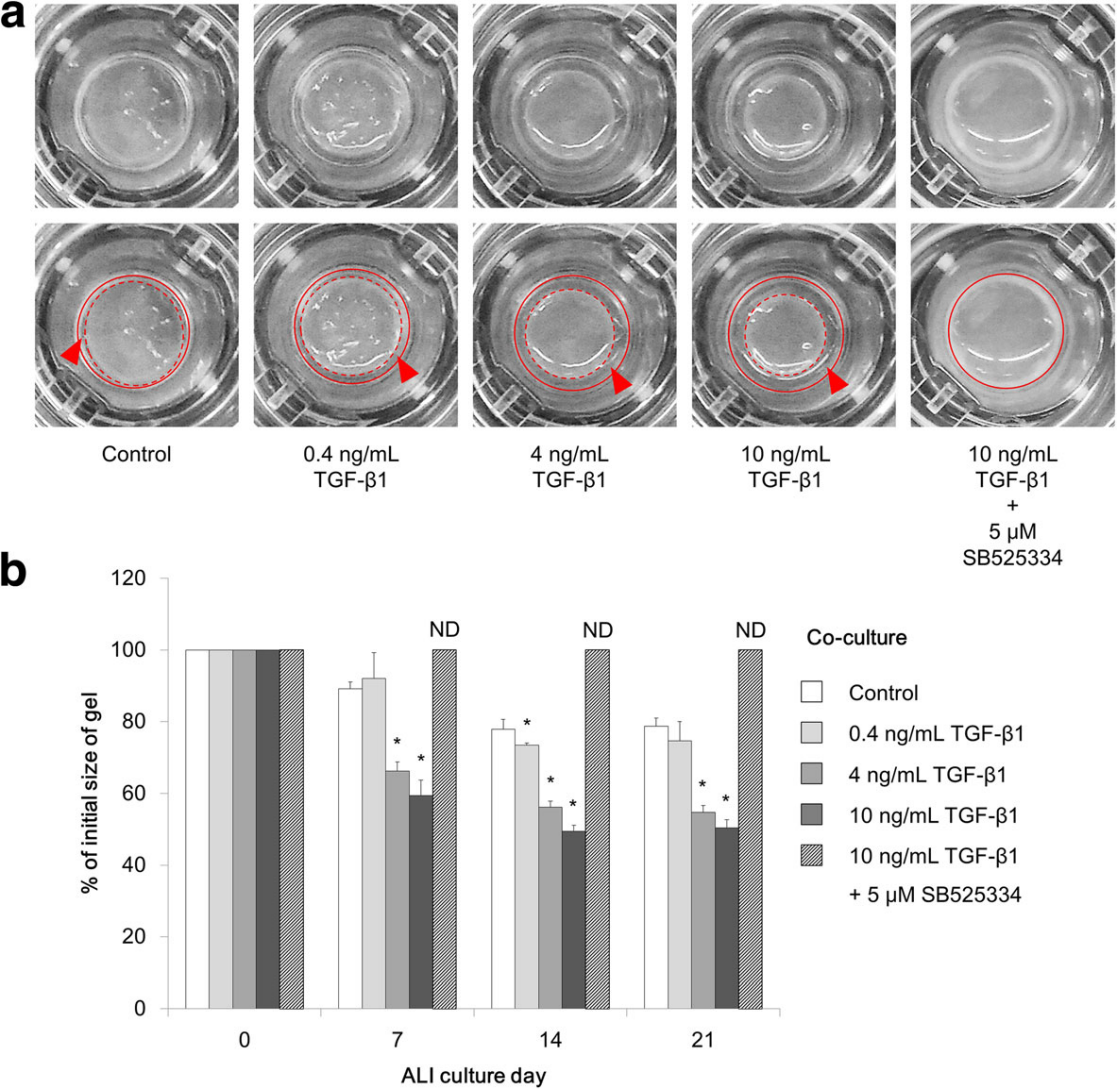
Quantitative analysis of collagen gel contraction in the co-culture model stimulated with TGF-β1. **a** Images of the air-liquid interface co-culture models stimulated with TGF-β1 on culture day 21. The edge of the cell culture insert (red line) and the edge of the collagen gel matrices (red dotted line) are shown in the images on the second row. Arrowheads indicate gaps between collagen gel and the wall of the cell culture insert. **b** Quantification of collagen gel contraction. Images were obtained on culture days 0, 7, 14, and 21; collagen gel contraction was quantified. The results represent the percentage of the initial gel size. Data are shown as means ± SD of triplicate inserts. *p < 0.05 (Dunnett’s test against the control at each time point). ND, not detected

The co-culture human bronchial tissue model with an epithelial cell layer and collagen-embedded fibroblast layer was stimulated with TGF-β1 for 21 days under ALI culture conditions (Fig. 1a). Collagen gel contraction was observed in the untreated control on ALI culture days 7, 14, and 21 (Fig. 1b). Gel contraction was significantly enhanced by stimulation with 4 or 10 ng/mL TGF-β1, compared with contraction in the the untreated control at each time point (*p* < 0.05). The collagen gel was clearly detached from the wall of the cell culture insert on ALI culture day 21 following stimulation with 4 or 10 ng/mL TGF-β1 (Fig. 1a, arrowheads). When the co-culture model was stimulated with 10 ng/mL TGF-β1 with simultaneous inhibition of TGF-β receptor type I by 5 μM SB525334, gel contraction was not detectable (Fig. 1b).

### Histological changes induced by TGF-β1 stimulation

**Fig. 2:**
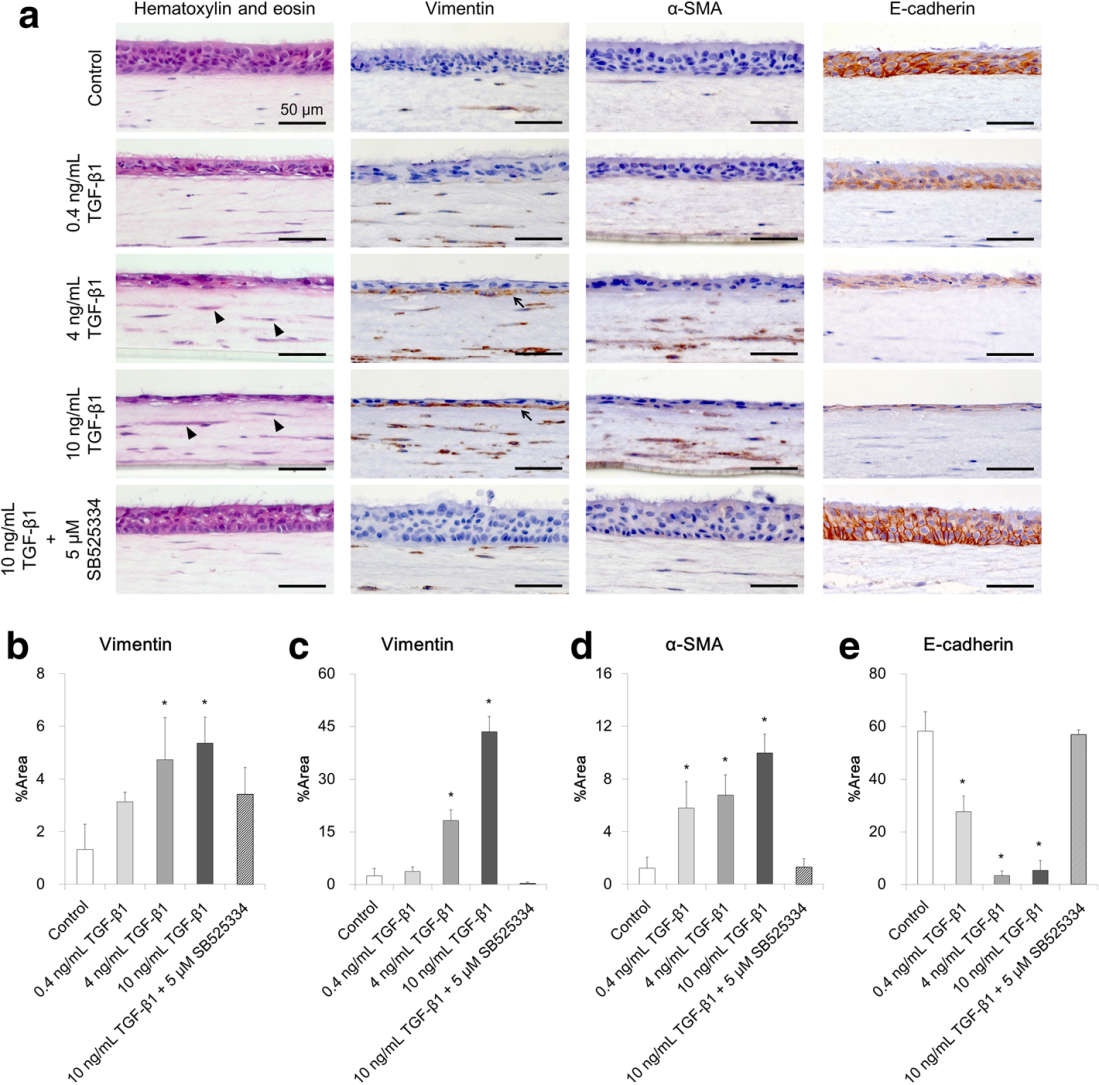
Histological analysis of epithelial and mesenchymal cells on culture day 21. **a** Hematoxylin and eosin staining, vimentin immunostaining, α-SMA immunostaining and E-cadherin immunostaining. Arrowheads indicate heavily stained fibroblast cells in the mesenchymal layer following TGF-β1 stimulation. Arrows indicate vimentin-positive basal cells in the epithelial layer following TGF-β1 stimulation. Scale bar: 50 μm. **b** Percentage of the vimentin-positive area in the mesenchymal layer of the tissue section. **c** Percentage of the vimentin-positive area in the epithelial layer of the tissue section. **d** Percentage of the α-SMA-positive area in the mesenchymal layer of the tissue section. **e** Percentage of the E-cadherin positive area in the epithelial layer of the tissue section. All data are shown as means ± SD of triplicate inserts. *p < 0.05 (Dunnett’s test against the control)

To further understand the effects of TGF-β1 on our co-culture model, we performed hematoxylin and eosin staining on histological sections of tissues collected on ALI culture day 21. We found effects of TGF-β1 on the cellular morphology and increased numbers of elongated HBECs (Fig. 2a). In accordance with this increase, a concentration-dependent decrease in the thickness of the epithelial layer was observed following stimulation with TGF-β1 (Fig. 2a). This decrease was prevented by the addition of 5 μM SB525334. In the mesenchymal collagen layer, we found an increase in heavily stained fibroblasts (arrowheads in Fig. 2a). Next, we performed immunostaining to characterize the changes observed in the sections stained with hematoxylin and eosin. We stained for vimentin as a mesenchymal marker, and found a significant increase in vimentin-positive fibroblasts in the mesenchymal layer following stimulation with 4 or 10 ng/mL TGF-β1 (*p* < 0.05) (Fig. 2a and b). Vimentin expression was also confirmed by TGF-β1 stimulation in the basal cells of the epithelial layer (Fig. 2a, arrows). The percentage of the vimentin-positive area in the epithelial layer increased significantly following stimulation with 4 or 10 ng/mL TGF-β1 (*p* < 0.05) (Fig. 2c). Immunostaining was also performed with an anti-α-SMA antibody to confirm the presence of myofibroblasts. Intracellular expression of α-SMA in fibroblasts increased significantly following TGF-β1 stimulation (0.4, 4, or 10 ng/mL; *p* < 0.05) (Fig. 2a and d). This increase was suppressed by blocking TGF-β1 signaling with 5 μM SB525334. We also performed immunostaining with an anti-E-cadherin antibody to confirm the epithelial status. E-cadherin is adhesion molecule that plays a major role in the maintenance of epithelial intercellular junctions. In contrast with the increased vimentin expression (Fig. 2a and c), we observed a decrease of E-cadherin in the epithelium following stimulation with TGF-β1 (Fig. 2a and e).

We also analyzed the effect of TGF-β1 on the differentiation of bronchial epithelial cells. We used acetylated α-tubulin as a ciliated cell marker, MUC5AC as a goblet cell marker, and CK5 as a basal cell marker. These differentiation markers were found to be expressed under control conditions (Additional file 1: Figure S1), following stimulation with TGF-β1, and even when blocking TGF-β1 signaling by 5 μM SB525334 (Additional file 1: Figure S1).

### Effect of TGF-β1 on expression of MMPs and TIMPs

**Fig. 3:**
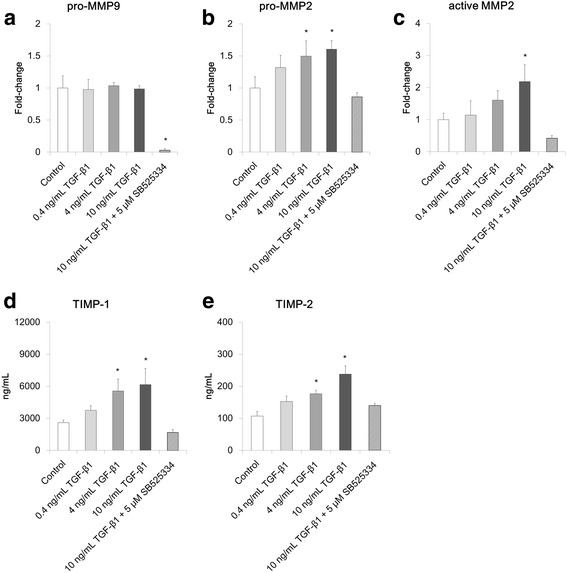
Analysis of matrix metalloproteinases (MMPs) and tissue inhibitors of metalloproteinase (TIMPs) in co-culture model. Quantitative analysis of band density in gelatin zymography of medium collected on culture day 21: Pro-MMP-9 (**a**), pro-MMP-2 (**b**), and active MMP-2 (**c**). The results represent fold changes relative to the control. Concentrations of TIMP-1 (**d**) and TIMP-2 (**e**) in medium collected on culture day 21. All data are shown as means ± SD of triplicate inserts. *p < 0.05 (Dunnett’s test against the control)

In addition to collagen gel contraction, altered ECM homeostasis is a factor in the airway remodeling process. We therefore analyzed the expression levels of proteolytic MMP-2 and MMP-9 in the culture medium of co-culture model collected on ALI culture day 21 by gelatin zymography. Pro-MMP-9 (92 kDa), pro-MMP-2 (72 kDa), and active MMP-2 (62 kDa) were detected (see co-culture data in Additional file 2: Figure S2); expression levels of each MMP were quantified as the fold change to the control (Fig. 3a–c). Expression of pro-MMP-9 in the co-culture model did not change after stimulation with TGF-β1 (Fig. 3a). However, pro-MMP-9 expression was suppressed significantly when TGF-β1 signaling was blocked with 5 μM SB525334 (*p* < 0.05). Expression of pro-MMP-2 and active MMP-2 in the co-culture model increased in a concentration-dependent manner following stimulation with TGF-β1, and significant increases were detected following stimulation with 10 ng/mL TGF-β1 (*p* < 0.05) (Fig. 3b and c). This increase in expression was blocked by the addition of 5 μM SB525334.

We also analyzed the concentrations of natural MMP inhibitor TIMPs in the co-culture medium on ALI culture day 21. Levels of TIMP-1 increased significantly following stimulation with 4 or 10 ng/mL TGF-β1 (*p* < 0.05) (Fig. 3d). Secretion of TIMP-1 following stimulation with 10 ng/mL TGF-β1 was suppressed to control levels by the addition of 5 μM SB525334. Secretion of TIMP-2 showed a similar trend, and increased significantly in response to TGF-β1, compared with levels in the control (*p* < 0.05) (Fig. 3e).

**Fig. 4:**
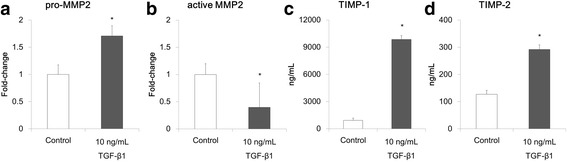
Analysis of matrix metalloproteinases (MMPs) and tissue inhibitors of metalloproteinase (TIMPs) in fibroblast mono-culture model. Quantitative analysis of band density in gelatin zymography of medium collected on culture day 21: Pro-MMP-2 (**a**), and active MMP-2 (**b**). The results represent fold changes relative to the control. Concentrations of TIMP-1 (**c**) and TIMP-2 (**d**) in medium collected on culture day 21. All data are shown as means ± SD of triplicate inserts. *p < 0.05 (Student’s *t*-test)

To confirm the role of epithelial-mesenchymal interaction in ECM homeostasis in the co-culture model, a mono-culture model with lung fibroblast cells was prepared. The fibroblast mono-culture model was stimulated with 10 ng/mL of TGF-β1, MMP secretion was analyzed with gelatin zymography. Unlike findings in the co-culture model, pro-MMP-9 expression was very low in the mono-culture even with TGF-β1 stimulation (see fibroblast mono-culture data in Additional file 2: Figure S2). Similarly to secretion in the co-culture model, pro-MMP-2 secretion in the mono-culture model increased significantly following stimulation with TGF-β1 (*p* < 0.05) (Fig. 4a). However, levels of active MMP-2 did not increase with TGF-β1 stimulation (Fig. 4b). Secretion of TIMPs in the mono-culture model stimulated with TGF-β1 was also analyzed; a significant increase in TIMP-1 and TIMP-2 levels was observed (*p* < 0.05) (Fig. 4c and d).

### Effect of TGF-β1 on ECM-related genes

**Fig. 5:**
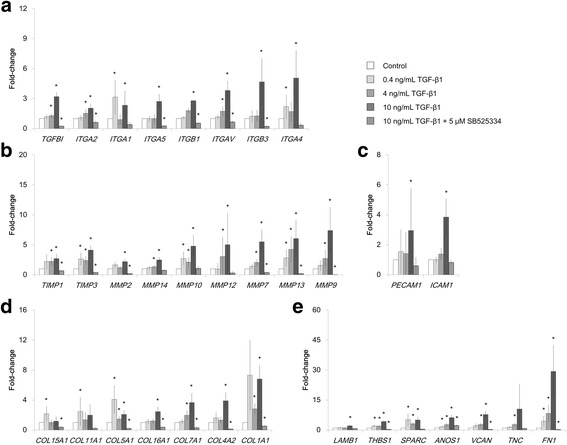
Increased expression of extracellular matrix-related genes following TGF-β1 stimulation in co-culture model on culture day 21. Expression levels of 84 genes were analyzed using PCR; 33 significantly upregulated genes are presented in the graphs. **a** TGF-β1 and integrins; **b** tissue inhibitors of matrix metalloproteinase (TIMPs) and matrix metalloproteinases (MMPs); **c** adhesion molecules; **d** collagens; **e** extracellular matrix glycoproteins and proteoglycans. All data are shown as means ±95% confidence intervals of triplicate inserts. *p < 0.05 (Student’s *t*-test against the control)

We analyzed the expression of ECM-related genes in the co-culture model on ALI culture day 21. Among the 84 genes analyzed, the expression of 33 genes increased significantly following stimulation with TGF-β1 (*p* < 0.05) (Fig. 5). These genes were categorized as follows: TGF-β and integrins (Fig. 5a), TIMPs and MMPs (Fig. 5b), adhesion molecules (Fig. 5c), collagens (Fig. 5d), and ECM glycoproteins and proteoglycans (Fig. 5e). The expression of most of these genes was upregulated in a concentration-dependent manner by TGF-β1 stimulation. These increases were suppressed to control levels or lower by the addition of 5 μM SB525334 (*p* < 0.05) (Fig. 5a–e). The top five genes upregulated following treatment with 10 ng/mL TGF-β1 in the co-culture model were *FN1* (29.3-fold change; Fig. 5e), *TNC* (10.5-fold change; Fig. 5e), *VCAN* (7.7-fold change; Fig. 5e), *MMP9* (7.38-fold change; Fig. 5b), and *COL1A1* (6.8-fold change; Fig. 5d).

**Fig. 6:**
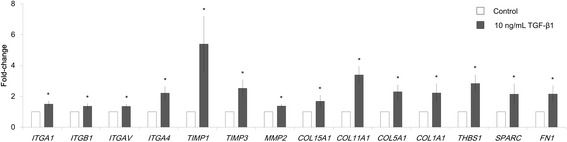
Increased expression of extracellular matrix-related genes following TGF-β1 stimulation in fibroblast mono-culture model on culture day 21. The expression levels of 84 genes were analyzed using PCR; 14 genes were commonly up-regulated in both co- and mono-culture models with 10 ng/mL TGF-β1 stimulation. The expression of these 14 genes in a mono-culture model is summarized graphically. Data are shown as means ±95% confidence intervals of triplicate inserts. *p < 0.05 (Student’s *t*-test against the control)

We also found that 14 of the 33 genes were upregulated in the fibroblast mono-culture model following stimulation with 10 ng/mL TGF-β1 (Fig. 6). However, the level of fold increase of several genes was lower in the mono-culture model compared with the co-culture model. For example, *FN1* expression showed a 29.3-fold change in the co-culture model after stimulation with 10 ng/mL TGF-β1 (Fig. 5e), compared with only a 2.2-fold change in the mono-culture model (Fig. 6).

### Histological analysis of the ECM protein expression pattern

**Fig. 7:**
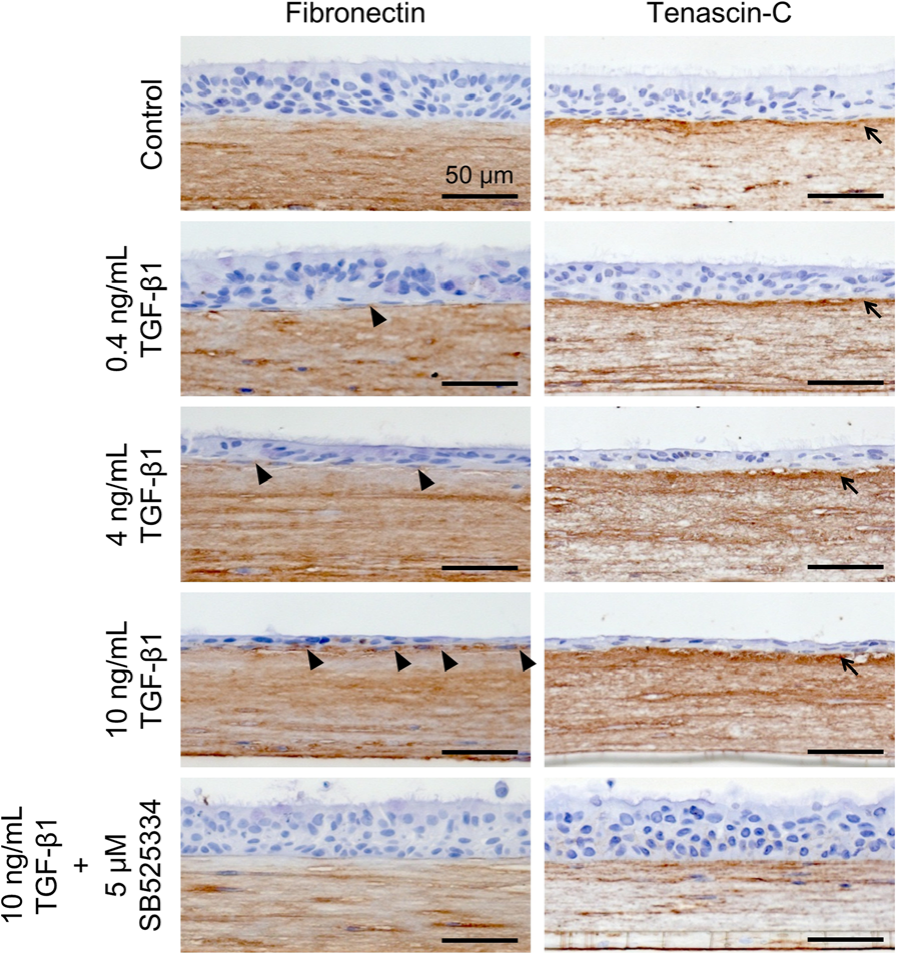
Histological analysis of extracellular matrix protein expression on culture day 21. Tissue sections were immunostained with anti-fibronectin and anti-tenascin-C antibodies. Arrowheads point to fibronectin-positive basal cells in the epithelial layer following TGF-β1 stimulation. Arrows indicate strong expression of tenascin-C in the sub-epithelial basement membrane following TGF-β1 stimulation. Scale bar: 50 μm

As *FN1* and *TNC* expression was markedly upregulated (Fig. 5e), we analyzed the expression pattern of proteins encoded by these genes in the co-culture. The results revealed fibronectin expression mainly across the mesenchymal layer (Fig. 7). However, we also found fibronectin-positive basal cells in the epithelial layer following TGF-β1 stimulation (Fig. 7, arrowheads). Tenascin-C was also expressed across the mesenchymal layer, and the staining appeared to become denser with higher TGF-β1 concentrations. Strong expression of tenascin-C was observed in the sub-epithelial basement membrane of the co-culture model (Fig. 7, arrows). Expression decreased as TGF-β signaling was suppressed by the addition of 5 μM SB525334.

## Discussion

The development of an in vitro lung model is essential to design new therapies for various lung diseases. Recent advances in bioengineering technology have enabled the generation of lung organoid models with matrigels and biocompatible alginate beads [19, 20]. One of the novelties of these models is the co-culture of multiple types of cells (such as fibroblasts, epithelial cells, and endothelial cells). Here, we report another type of co-culture model involving the ALI culture of HBECs on collagen gel matrices with fibroblasts.

The collagen gel contraction assay is well-established, and multiple studies have shown that TGF-β signaling can enhance the contractility of gels in submerged cultures [4–6]. As our co-culture model has a collagen-embedded fibroblast layer, we examined whether stimulation by TGF-β1 could promote gel contraction in an ALI culture. The cells were cultured for 21 days under ALI conditions to induce the mucociliary differentiation of HBECs [21]; TGF-β1 stimulation was conducted during the ALI culture period. Collagen gel contraction was detected in the untreated (control) culture, and TGF-β1 treatment enhanced contraction at each time point during the ALI culture period. We also found that gel contraction was suppressed by the TGF-β type I receptor blocker SB525334, which inhibits activin receptor-like kinase (ALK) 5-mediated Smad2/3 phosphorylation [22, 23]. This finding suggests that the effect of TGF-β1 on gel contraction in our experiment was mediated through the TGF-β1/ALK5 pathway, and is supported by a report that Smad3-mediated TGF-β1 signaling is necessary for collagen gel contraction [24].

To elucidate the mechanisms behind the gel contraction, we analyzed histological changes in our in vitro EMTU. We found increased numbers of elongated HBECs by hematoxylin and eosin staining. Induction of epithelial-mesenchymal transition (EMT) is one of the most prominent effects of TGF-β in various tissues [25, 26], and HBECs cultivated under ALI conditions have been reported to exhibit an elongated shape with EMT characteristics following stimulation with TGF-β [27]. Epithelial cells show decreased expression of the epithelial marker E-cadherin when undergoing EMT [28]. In our co-culture model, we confirmed a decrease in E-cadherin expression in the epithelial layer following TGF-β1 stimulation. We also found an increase in basal cells positive for the mesenchymal marker vimentin in the epithelial layer and in vimentin-positive fibroblasts in the mesenchymal layer. These results suggest that TGF-β1 promotes the proliferation of fibroblasts in the mesenchymal layer, and that basal cells in the epithelial layer undergoing EMT could be a source of fibroblasts. In addition, we found that the fibroblasts were α-SMA-positive myofibroblasts. Induction of contractile myofibroblasts is considered important in collagen gel contraction [6, 29]. Thus, a concentration-dependent induction of myofibroblasts in the mesenchymal layer may have promoted the gel contraction we observed. Histological analysis indicated that TGF-β1 induced EMT in the epithelial layer and myofibroblasts in the mesenchymal layer. This suggests that our culture model successfully reproduced multiple events in the EMTU simultaneously.

The effect of TGF-β1 on the epithelial layer was also analyzed using antibodies against markers of ciliated cells, goblet cells, and basal cells, which are all present in a differentiated bronchial epithelium. We found the existence of all three cell types with or without TGF-β1 stimulation. This suggests that the effect of TGF-β1 on bronchial epithelial differentiation is limited. Moreover, we even found that the differentiation markers were expressed when TGF-β1 signaling was blocked by SB525334. This is consistent with a previous study in which mice with a deletion of *ALK5* in the lung showed a differentiation of ciliated cells similar to control mice [30].

Apart from myofibroblast induction, an increase in ECM protein deposition is an important event in the airway remodeling process induced by TGF-β1 [31], and both MMPs and TIMPs are involved. MMPs digest ECM components and are considered important mediators of tissue remodeling [32, 33]. MMPs are regulated by their natural inhibitors, TIMPs [34]. The MMP/TIMP balance is a critical factor in controlling the overall proteolytic activity in various tissues [35–37]. We measured the expression levels of MMP-9 and MMP-2 in the co-culture model on culture day 21 by gelatin zymography, as well as the levels of TIMP-1 (main inhibitor of MMP-9) and TIMP-2 (main inhibitor of MMP-2). TGF-β1 stimulation promoted secretion of pro-MMP-2 and active MMP-2 in a concentration-dependent manner (Fig. 3b and c). Levels of pro-MMP-9 secreted into the medium did not change with TGF-β1 stimulation (Fig. 3a). However, the involvement of TGF-β signaling in pro-MMP-9 secretion was seen when TGF-β signaling was blocked with SB525334. In line with the increases observed in MMP expression, secretion of TIMP-1 and TIMP-2 also increased (Fig. 3d and e). As both MMPs and TIMPs were upregulated, the MMP/TIMP balance could be maintained in the co-culture model even following stimulation with TGF-β1. In a study with a fibroblast mono-culture model, we found that pro-MMP-9 and active MMP-2 were not induced by TGF-β1 (Fig. 4b and Additional file 2: Figure S2), while TIMP-1 and TIMP-2 were (Fig. 4c and d). These findings suggest that epithelial cells were necessary for secretion of pro-MMP-9 and active MMP-2 in the co-culture, and proteolytic activity of these MMPs was controlled by the TIMPs secreted from mesenchymal fibroblasts. Thus the concept that epithelial-mesenchymal interaction is important in controlling ECM protein degradation is supported.

To further characterize the effects of TGF-β1 on ECM deposition in the co-culture model, we examined expression changes in genes related to ECM production. Thirty-three genes were significantly upregulated. We surmise that these genes are regulated by the TGF-β1/ALK5 pathway, as the TGF-β1 blocker SB525334 suppressed increases in their expression. Integrins are transmembrane receptors that exert various effects on tissue remodeling and contribute to EMTU homeostasis [38, 39]. The integrin families upregulated in our assays have been shown to affect tissue remodeling by mechanisms such as myofibroblast induction and activation of TGF-β [40]. Consistent with the findings for TIMPs and MMPs, gene expression of *MMP2*, *MMP9*, and *TIMP1* was regulated by TGF-β1 signaling. In addition, we found increases in the expression of *MMP14*, *MMP10*, *MMP12*, *MMP7*, and *MMP13*. Higher levels of the MMPs encoded by those genes have been reported in idiopathic pulmonary fibrosis patients and in a murine model of lung disease, and these increases may play various roles in airway tissue remodeling [41]. We also found increased expression of genes encoding various collagens and ECM glycoproteins and proteoglycans, which could be substrates for the proteolytic activity of MMPs. Expression of these genes suggests the promotion of ECM production.

We obtained gene expression data from both the co-culture model and the fibroblast mono-culture model (Figs. 5 and 6). We found that 14 of the 33 genes whose expression was increased following stimulation with TGF-β1 were also up-regulated in the fibroblast mono-culture model after TGF-β1 simulation. The reduced number of up-regulated genes in the fibroblast mono-culture model suggests that co-culture conditions are necessary for the induction of several genes. This suggests an involvement of HBECs in the remodeling process induced by TGF-β1.

We found that *FN1* and *TNC* expression increased notably following stimulation by 10 ng/mL TGF-β1 in the co-culture model. The expression pattern in the lung of proteins encoded by these genes, fibronectin and tenascin-C, was reported to be related to lung disease [42, 43]. Histological analysis determined that these proteins were expressed across the mesenchymal layer. However, we found an increase in fibronectin-positive basal cells following TGF-β1 stimulation. In addition to vimentin expression, fibronectin expression in basal cells is considered one aspect of EMT [44]. We also found strong expression of tenascin-C in the basement membrane of the co-culture model, in a pattern consistent with in vivo expression [45]. The expression of both fibronectin in basal cells and tenascin-C in the sub-epithelial region decreased following treatment with the TGF-β1 blocker SB525334, indicating that the TGF-β1/ALK5 pathway is involved in ECM deposition in bronchial tissues. Reproduction of the sub-epithelial region in vitro is possible when epithelial cells are directly co-cultured with collagen-embedded mesenchymal cells, and fibrotic changes in this region (i.e. sub-epithelial fibrosis) are important steps in the pathogenesis of asthma [46, 47]. Hence, our in vitro EMTU model could be useful in elucidating how changes in the sub-epithelial region are induced by the interaction between epithelial cells and mesenchymal layer.

One of the mechanisms in the epithelial–mesenchymal interaction is paracrine signaling involving growth factors and cytokines [15–17]. The interaction is also mediated by integrins, which could be used as therapeutic targets in airway hyperresponsiveness and remodeling [48]. Various studies suggest that the ECM composition affects cell signaling, cell proliferation, apoptosis, and EMT in the epithelial cells through adhesion through integrins [49–51]. Our co-culture model demonstrated changes in ECM protein and integrin gene expression following TGF-β1 stimulation. Thus, our model has the potential to recapitulate cell–cell and cell–ECM interactions mediated through adhesion by integrins, which is possible when epithelial cells are directly co-cultured with a mesenchymal layer.

## Conclusion

We developed a co-culture model of human bronchial tissue that enables direct interactions to occur between epithelial cells, mesenchymal cells, and their ECM components. This model successfully reproduced multiple events including EMT in the epithelial layer, myofibroblast accumulation in the mesenchymal layer, and ECM deposition in the airway remodeling process induced by TGF-β1. These events were blocked with SB525334. Thus, our model could be useful in the study of airway remodeling in vitro, as well as drug testing. Interestingly, the effect of TGF-β1 was different in the co-culture model compared with the fibroblast mono-culture model. In the future, we plan to introduce a mono-culture model of HBECs to our experimental design, which will enable us to investigate differences in the response to TGF-β1 stimulation among these three culture models to clarify the effect of co-culture in more detail. Moreover, treatment with target-specific small interfering RNAs or antibodies should help determine the mechanism underlying epithelial–mesenchymal cross-talk in EMTU components. The application of cells from patients will also aid an investigation of epithelial–mesenchymal cross-talk in disease progression.

## Additional files

Additional file 1: Figure S1. Histological analysis of differentiation markers of bronchial epithelium on culture day 21. Tissue sections were immunostained with an anti-acetylated α-tubulin antibody (ciliated cell marker), anti-MUC5AC antibody (goblet cell marker), and anti-CK5 antibody (basal cell marker). Scale bar: 50 μm. (JPEG 2811 kb)

Additional file 2: Figure S2. Gelatin zymography of culture medium from the co-culture model and fibroblast mono-culture model collected on culture day 21. (JPEG 669 kb)

## Abbreviations

ALI: Air-liquid interface
ALK: Activin receptor-like kinase
CK: Cytokeratin
DAB: Diaminobenzidine
ECM: Extracellular matrix
EMT: Epithelial-mesenchymal transition
EMTU: Epithelial-mesenchymal trophic unit
HBEC: Human bronchial epithelial cell
MMP: Matrix metalloproteinase
TGF: Transforming growth factor
TIMP: Tissue inhibitor of metalloproteinase
