# Supplementary figures and images for: A 3D epithelial–mesenchymal co-culture model of human bronchial tissue recapitulates multiple features of airway tissue remodeling by TGF-β1 treatment
Source: Respir Res. 2017 Nov 22;18:195. doi: 10.1186/s12931-017-0680-0 (PMC5700468; doi:10.1186/s12931-017-0680-0)

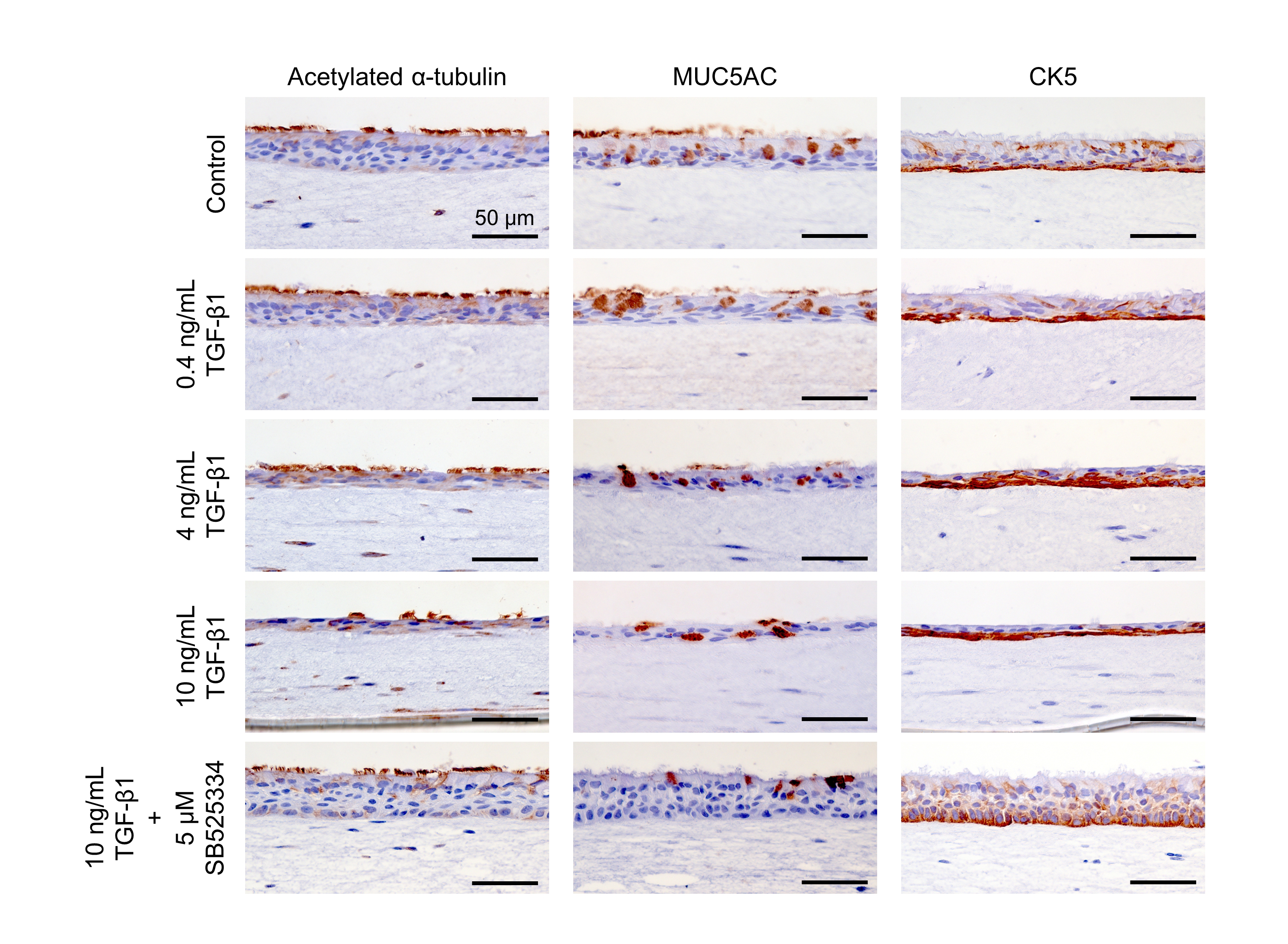

Supplement: Supplementary file 1 — Histological analysis of differentiation markers of bronchial epithelium on culture day 21. Tissue sections were immunostained with an anti-acetylated α-tubulin antibody (ciliated cell marker), anti-MUC5AC antibody (goblet cell marker), and anti-CK5 antibody (basal cell marker). Scale bar: 50 μm. (JPEG 2811 kb) [file 12931_2017_680_MOESM1_ESM.jpg]

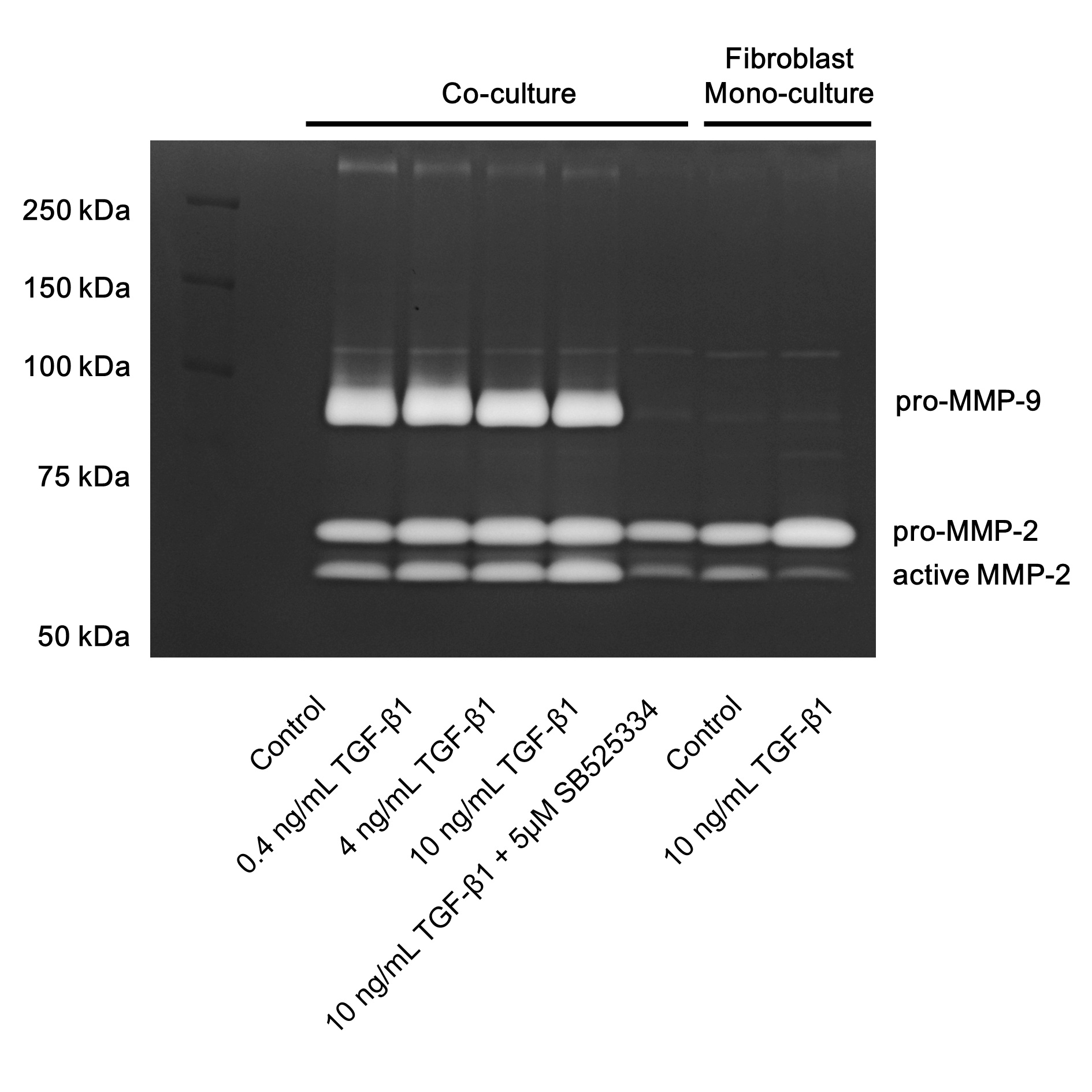

Supplement: Supplementary file 2 — Gelatin zymography of culture medium from the co-culture model and fibroblast mono-culture model collected on culture day 21. (JPEG 669 kb) [file 12931_2017_680_MOESM2_ESM.jpg]
